# Lignocellulose-based analytical devices: bamboo as an analytical platform for chemical detection

**DOI:** 10.1038/srep18570

**Published:** 2015-12-21

**Authors:** Chen-Meng Kuan, Roger L. York, Chao-Min Cheng

**Affiliations:** 1 Institute of Nanoengineering and Microsystems, National Tsing Hua University, Hsinchu 30013, Taiwan; 2 David H. Koch Institute for Integrative Cancer Research, Massachusetts Institute of Technology, 77 Massachusetts Avenue, Cambridge, MA 02139, USA; 3 Department of Anesethesiology, Boston Children’s Hospital, 300 Longwood Avenue, Boston, MA 02115, USA; 4 Institute of Biomedical Engineering, National Tsing Hua University, Hsinchu 30013, Taiwan

## Abstract

This article describes the development of lignocellulose-based analytical devices (LADs) for rapid bioanalysis in low-resource settings. LADs are constructed using either a single lignocellulose or a hybrid design consisting of multiple types of lignocellulose. LADs are simple, low-cost, easy to use, provide rapid response, and do not require external instrumentation during operation. Here, we demonstrate the implementation of LADs for food and water safety (i.e., nitrite assay in hot-pot soup, bacterial detection in water, and resazurin assay in milk) and urinalysis (i.e., nitrite, urobilinogen, and pH assays in human urine). Notably, we created a unique approach using simple chemicals to achieve sensitivity similar to that of commercially available immunochromatographic strips that is low-cost, and provides on-site, rapid detection, for instance, of *Eschericia coli* (*E. coli*) in water.

The use of point-of-care (POC) diagnostics to make on-site clinical decisions and develop home tests is an important strategy for disease management. The development of POC diagnostics benefits both third-world countries and developed countries due to the following advantageous characteristics: i) affordability; ii) high degrees of sensitivity and specificity; iii) ease of use; iv) rapidness; v) robustness; and, vi) functionality independent of supporting equipment for disease diagnosis^1^,^2^. The development of POC diagnostics has imparted a transformative effect on healthcare systems throughout the world^3^. Of the current developments in POC diagnostics, those leveraging matrix-based materials, such as paper, thread, cloth, and cotton, contribute most to low-cost diagnostic platforms^4^,^5^,^6^,^7^,^8^,^9^,^10^. In particular, paper-based analytical devices (PADs), categorized by the use of dipsticks and both lateral and flow-through assays, have become a standard platform for low-cost diagnostics^11^,^12^,^13^,^14^,^15^,^16^,^17^,^18^,^19^. The development of PADs leverages well-defined hydrophobic patterns to build specific target zones, or reaction areas, for chemical analyses^20^. This is commonly accomplished by photolithography, CO_2_ laser cutting, printing with polymeric materials or waxes, wax dipping, inkjet etching, plasma treatment, drawing by hand with reagent pens or pencils, melt-and-mold fabrication, and initiated chemical vapor deposition (iCVD)^4^,^18^,^19^,^20^,^21^,^22^,^23^,^24^,^25^,^26^,^27^,^28^,^29^. Some of the materials used to build hydrophobic boundaries, however, are not suitable for assays that require organic solvents (e.g., alcohol assay) – organic liquids can dissolve poly(styrene) and/or waxes, and cause swelling in polydimethylsiloxane (PDMS)^30^. In addition to disease diagnostics, the World Health Organization (WHO) has also viewed water quality, food security, environment, and energy as important aspects of global health in recent years^31^. For example, lower respiratory tract infections and chronic obstructive pulmonary disease are associated with deleterious air quality, and diarrheal diseases (the third cause of mortality in developing countries) are caused by unsafe water or food^31^. The WHO points out that the simplest and most cost-efficient strategy to alleviate the risks of food hazards is to identify, assess, and monitor foodborne dangers before consumption^32^.

PDMS-based microfluidic systems are another common technology for POC diagnostics. PDMS-based microfluidic systems provide an alternative route to analyze biological information or resemble microelectromechanical systems (MEMS), typically made up of silicon and glass, on different substrates^33^,^34^. PDMS-based systems impart several advantages for biochemical applications (e.g., single cell, DNA, and RNA analyses and drug screening or even protein crystallization) as follows^33^,^35^,^36^,^37^,^38^,^39^: i) they can be conveniently replicated through soft lithography; ii) PDMS is transparent, biocompatible, and a gas permeable material; iii) they can provide a route to integrate current technologies into a compact device (lab-on-a-chip); and, iv) they provide reagent- and sample-saving qualifications for liquid handling. In most cases, the fabrication of PDMS-based microfluidic systems relies on soft lithography following photolithography and oxygen plasma treatment on the surface of PDMS for device bonding^40^. Moreover, fluidic pumps, valves, and mixer are essential for controlling as well as mixing laminar fluid flows in microfluidic channels, and are commonly accompanied by a reader or an electronic recorder to observe results^41^,^42^. These sophisticated experimental requirements render the use of such devices more difficult for home tests. To overcome these shortcomings, the use of microfluidic PADs has been proposed.

The main objective of this article is to demonstrate that analytical devices can be built directly from easily accessible materials (i.e., wood and bamboo stirrers) impregnated with colorimetric indicators to analyze food and water safety and complete urinalysis. The physical fabrication of lignocellulose-based analytical devices (LADs) requires no more than mechanical processing (i.e., drilling), and is not dependent on chemical manufacturing processes. Although we and others have examined lignocelluloses as components for biofuels and solar cells and as potential application platforms for water security and POC diagnostics, the intricate development of LADs has not yet been unveiled^31^,^43^,^44^,^45^,^46^. Lignocelluloses are composed of plant cell walls whose major components are cellulose, hemicelluloses, and lignin that, together, form microfibrils. These microfibrils are organized into macrofibrils that provide structural stability to the plant and construct the vascular bundle for nutrient (phloem) and fluid transport (xylem)^46^. Lignocelluloses, i.e., bamboo and wood stirrers, provide several advantageous characteristics for use as analytical devices: (i) they are inexpensive (bamboo and wood stirrers from local retailers cost approximately USD 0.009/ea. and approximately USD 0.017/ea., respectively) and ubiquitous; (ii) they exploit natural one-dimensional capillary action to transport fluids along lignocellulosic channels without the demand for additional energy or instrumentation (e.g., pumps)^47^; (iii) they allow for simple fabrication into devices; (iv) they are resistant to dissolution or degradation by organic solvents^48^; (v) they provide for easy post-use disposal via burning or biodegradation; and, (vi) they are lightweight and easy to carry. Bamboo is an abundant plant in Asia where bamboo-derived industries are much more prevalent than they are in other continents^49^. We are convinced that the accumulated experiences from bamboo industries would benefit the development of LADs and facilitate large-scale LAD production.

This paper demonstrates the construction of both single-material (i.e., “bamboo”) lignocellulose-based analytical devices (SMLADs) and hybrid (i.e., “bamboo and wood”) lignocellulose-based analytical devices (HLADs). We fabricated SMLADs to demonstrate the simplicity of LADs; we then fabricated HLADs by adding another lignocellulose in order to provide greater flexibility and utility. Here, we show that these low-cost, robust devices can be used for nitrite, PMS-MTT, resazurin, urobilinogen, pH, and glucose assays.

## Results

### Lignocellulosic Physical Properties

**Figure 1:**
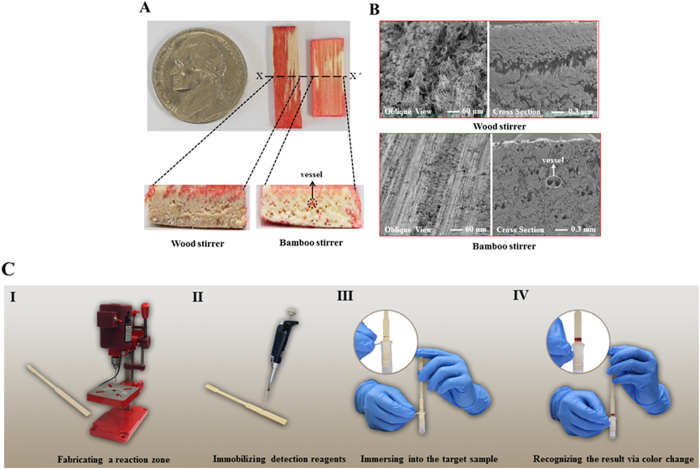
Wicking phenomena and detailed structure information of wood and bamboo stirrers. (**A**) Optical images show oblique and cross section views for wood and bamboo stirrers after wicking red dye solution (separating along the A to A’ direction). (**B**) Images show oblique and cross section views for wood and bamboo stirrers via SEM measurement. (**C**) Schematic of the fabrication process for single-material lignocellulose-based analytical devices: (I) creating a groove as a reaction zone on the surface of bamboo stirrer with a drill press, (II) immobilizing detection reagents (colorimetric reagents) with a micropipette into the reaction zone, (III) immersing the SMLADs into the target samples, (IV) recognizing the colorimetric results on the surface of reaction zone. (Photo credits: T.-L. Wang, C.-M. Kuan).

In an attempt to build LADs, we investigated wicking capability from some prospective materials (commercially available wood and bamboo stirrers). Because each stirrer has distinctive geometry and width or thickness as received from the manufacturer (i.e., they are not cut in any direction), we decided to define the wicking distance in terms of a height such that the total volume of material that wicks liquid is 250 mm^3^ (e.g., the wicking distance for a material with a larger base would be smaller than the wicking distance for a material with a smaller base). Table 1 shows the wicking results for six wood and four bamboo stirrers. The results indicate that bamboo stirrers wicked red dye solution at a rate approximately one order of magnitude higher than that of wood stirrers. Additionally, the wicking rates of the wood stirrers appear burdened by greater irreproducibility relative to the bamboo stirrers. This irreproducibility is influenced by relatively longer wicking duration. Results of the wicking test also show that dye (red) was prominently discovered on wood surface, and not wood core; by contrast, dye could be readily found in the internal portion of bamboo (via vessels—one of the common elements in a vascular bundle) as well as on bamboo surface (Fig. 1A). Figure 1B displays scanning electron microscope (SEM) images of the wood and bamboo stirrers (observed via oblique and cross sectional views). We discovered that external structures of bamboo and wood stirrers were comprised of soft fibers and hollow microchannels were only observed within bamboo stirrers. Considering the faster and more reproducible wicking rates of the bamboo stirrers, we choose to employ them as SMLADs for biochemical analyses.

We also examined the wicking rates of two paper strips (widths 0.7 cm and 0.3 cm) and one LAD (a bamboo stirrer, width 0.7) cm (Supplementary Figure 1) (Supplementary Movie 1). We used Whatman filter paper for PADs instead of nitrocellulose membrane. We, and others, have verified the availability of filter paper, especially Whatman filter paper No. 1 as the primary device substrate, in a number of assays^11^,^12^,^13^,^14^,^15^,^16^,^17^,^18^, while nitrocellulose membrane has generally been used for the development of immunochromatographic tests rather than common metabolic assays. The wicking distances for the two paper strips were approximately 1.5 cm (6 mm/sec), while the wicking distance for the LAD was approximately 3 cm (12 mm/sec) over the course of 25 seconds. Moreover, the wicking rates for different grades of chromatography paper from Whatman were in the range of 0.064–0.1 mm/sec^50^. For these reasons, we propose that bamboo is a suitable substrate to develop lateral flow-based assays in light of the relatively low barrier to mass transfer, in particular, for assays requiring long wicking distances or times.

Supplementary Figures 2 displays SEM images of purchased bamboo stirrers, and bamboo stirrers after treatment with methanol, 1-propanol, and acetone, respectively, over ten minutes. We found that the internal structure of treated bamboo stirrers was similar to that of the control group. This could allow for the use of LADs in assays that require organic solvents.

### Design of LADs

The simplest forms of LADs one can imagine are copies of lateral flow-based PADs. These LADs are one-dimensional, low-cost, and easy to operate. As a proof-of-concept, we choose to fabricate these devices, composed of bamboo stirrers, using only a drill press (fabrication time: 10 sec/ea.; fabrication cost: USD 0.01/ea.). This allowed us to demonstrate the capability of such devices while facilitating easy future entry into industrial manufacturing. Detailed LAD design and the operation process are provided in Fig. 1C.

**Figure 2:**
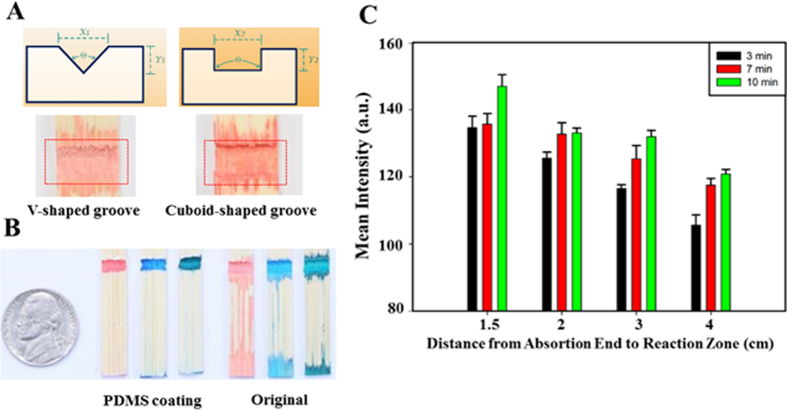
Investigation of reaction zone design and solution transportation for single material lignocellulose-based analytical devices. (**A**) Bamboo stirrer reaction zone design was examined to optimize reaction zone geometry. Immobilizing 3 μL red dye solution (5 mM allura red AC) into the V-shaped (θ = 120°, X1 = 3 mm, Y1 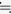 0.9 mm) and cuboid-shaped grooves (θ = 180°, X2 = 3 mm, Y2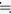 0.9 mm) showing that the V-shaped groove demonstrated more uniform red dye distribution. (**B**) Investigating the feasibility of internal channels. Treating external part of bamboo stirrers with PDMS (polydimethylsiloxane) to dramatically increase the influence of the internal channel. Dye tests (5 mM allura red AC, 10 mM disperse blue 14, 10 mM fast green) verified internal channel capability. (**C**) Investigating the correlation between mean color intensity (the amount of chemical concentration) and the position design of reaction zone and immersed time. We designed four reaction zone positions—1, 2, 3, and 4 cm (distances between absorption end and reaction zone). Next, we immersed SMLADs with different design positions into the red dye solution over several time periods (3, 7, and 10 minutes) before measuring and recording the results shown. Considering the demand of avoiding contamination in the detection samples, while still providing an ample colorimetric signal output, 3 cm and 7 minutes appear optimal for this implementation. (*N* = 10; mean intensity ± S.E.M.).

Figure 2A shows two experimental reaction zone geometries fabricated using a drill press: i) a V-shaped groove (θ = 120°); and, ii) a cuboid-shaped groove (θ = 180°), both following application of 3 μL of red dye solution (5 mM allura red AC) via a pipetman onto each reaction zone. The result indicates that a reaction zone in a V-shaped groove could provide stronger signal than one in a cuboid-shaped groove, which may be due to following reasons: i) red dye solution is distributed more uniformly when deposited into the V-shaped groove; and, ii) red dye solution deposited on vertical edges of cuboid-shaped groove could not be observed and measured. Hence, groove geometry has an influence on the visible spatial distribution of red dye solution within the reaction zone. Figure 2B compares the wicking properties of unmodified and PDMS-coated bamboo stirrers. This photograph shows that the aqueous dyes (5 mM allura red AC, 10 mM disperse blue 14, and 10 mM fast green) wicked via both the interior and exterior of the untreated bamboo stirrers, but only wicked via the interior of the PDMS coated stirrers. To explore the relationship between reaction zone position and detection performance, we examined the impact of using stirrer devices when the distance from absorption end to reaction zone was varied (Fig. 2C). These results indicate that the position of the reaction zone strongly influences the sensitivity of colorimetric assays and longer immersion times increased the amount of dye in the reaction zone regardless of reaction zone position. We consider the intensity difference between 7 and 10 minutes to be small, and the signal measured at 7 minutes to be sufficiently high for the experiments described here. As a result of these experiments, we chose to build the devices discussed in this paper with a reaction zone that was 3 cm from the absorption end, and a mean intensity of colorimetric assays measured after 7 minutes of immersion into solution (note that if the reaction zone is too close to the absorption end, the chemicals in the reaction zone were prone to spill into the sample being analyzed).

### SMLADs

**Figure 3:**
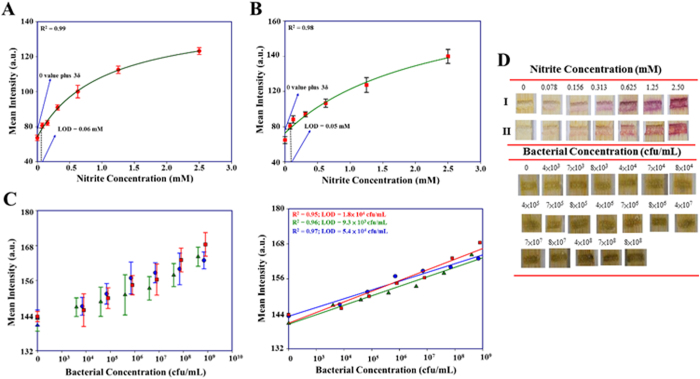
Lignocellulose-based analytical devices for food and water safety. All images of colorimetric results were obtained via a digital camera and analyzed via ImageJ software; these graphs represent the analysis for food safety (nitrite assay and bacterial detection in drinking water (PMS-MTT assay). The nitrite assay was conducted in deionized water (**A**) and hot-pot soup (**B**), and the establishment of a calibration curve under several concentrations—0, 0.078, 0.156, 0.013, 0.625, 1.25, and 2.5 mM—resulted. (deionized water: *N* = 26; mean intensity ± S.D.) (hot pot soup: *N* = 15; mean intensity ± S.D.) (**C**) The PMS-MTT assay was conducted in the conditions of three individual bacterial solution samples—4 × 10^8^, 7 × 10^8^, and 8 × 10^8^, each of which was serially diluted to five different concentrations of *E. coli* solution—(4 × 10^3^, 4 × 10^4^, 4 × 10^5^, 4 × 10^6^, and 4 × 10^7^), (7 × 10^3^, 7 × 10^4^, 7 × 10^5^, 7 × 10^6^, and 7 × 10^7^), and (8 × 10^3^, 8 × 10^4^, 8 × 10^5^, 8 × 10^6^, and 8 × 10^7^) —in drinking water. The three calibration curves were constructed via each diluted solutions. (*N* = 5; mean intensity ± S.D.) (**D**) Images display for nitrite (I: deionized water; II: hot-pot soup) and PMS-MTT assays.

**Figure 4:**
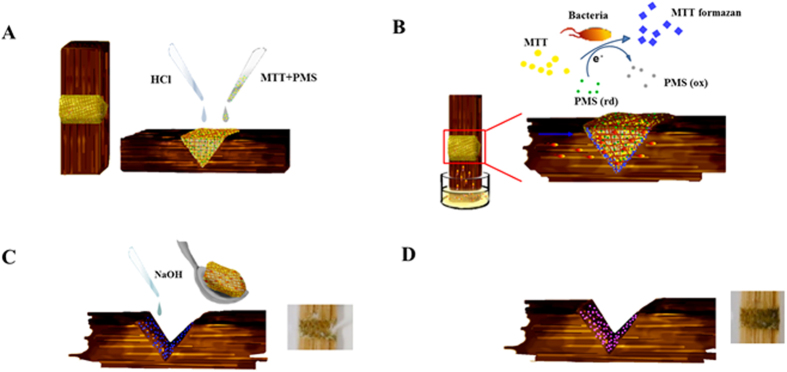
Schematic of the bacteria detection using lignocellulose-based analytical devices. (**A**) Immobilizing α-cellulose powder into the groove of SMLADs, and then adding 15.63 mM HCl solution on α-cellulose—HCl was used to eliminate hydroxyl group interference in α-cellulose on bacterial detection reagents. Five minutes were allowed for liquid to dry. Bacterial reaction agents were applied (3.26 mM PMS and 6.03 mM MTT) on α-cellulose following HCl treatment. (**B**) Bacterial detection was initiated by dipping the device into target samples for 5 minutes. (**C**) Removing α-cellulose and adding 31.25 mM NaOH, which could enhance colorimetric signals of the bacterial detection, to the groove of SMLADs. (**D**) Observing/recording colorimetric results via naked eye/a camera.

In regards to food safety, we demonstrate nitrite detections in deionized water and hot-pot soup (mainly containing chili, Chinese herbs, and pig fat) as well as bacterial detection in water using SMLADs. Nitrite is a common food additive in processed meat products (e.g., ham and hot dogs) that is used to inhibit bacterial growth and avoid meat spoilage. However, the use of nitrites may result in human health concerns, including cancer^51^. The World Health Organization (WHO) recommends the acceptable daily intake (ADI) for nitrite as 0–0.07 mg/kg body weight, i.e., a sixty-kg person should not take in more than 4.2 mg nitrite per day and should avoid having hot-pot soup with a nitrite concentration over 0.1 mM (using 1L hot-pot soup as a calculation standard)^52^. The limits of detection (LODs) of our nitrite assay in deionized water and hot-pot soup were 0.06 mM and 0.05 mM, respectively (Fig. 3A,B). According to the WHO, approximately 3.1% of annual deaths (1.7 million) and 3.7% of disability-adjusted life years worldwide (54.2 million) are associated with unsafe water, sanitation, and hygiene^53^. Growing bacteria on agar plates is the gold standard for determining bacterial presence, species, and number, but some limitations are rooted in this method: (i) it is time-consuming (commonly requiring more than one day); (ii) the preparation for agar plates themselves requires at least 1 hour; (iii) disposing of the plastic plates after each experiment is not eco-friendly; and, (iv) an incubator is required. In our experiment, we developed a unique combination of LADs and a PMS-MTT (phenazine methosulfate [PMS], 3-[4,5-dimethylthiazol-2-yl]-2,5-diphenyltetrasodium bromide [MTT]) assay to quantify the presence of bacteria in water (Figs 3C and 4) (Supplementary Figure 3, Movie 2), and compared our results with agar plate results (Supplementary Figure 3) to confirm the amounts of bacteria in each sample. We serially diluted standard *E. coli* solutions (4 × 10^8^, 7 × 10^8^, and 8 × 10^8^) to five different concentrations of *E. coli* in drinking water. The total analyzed time for this detection was less than 1 hour. Results indicate that our methods successfully detected distinct concentrations of bacteria in water and the linear trends observed in three different standard *E. coli* solutions were similar to each other, indicating reproducibility. The bacterial detection LODs were in the range of 1.8 × 10^4^–9.3 × 10^4^ cfu/mL (the levels of *E. coli* in running water must not exceed 10^2^ cfu/mL)^54^. We also used LADs to determine milk quality via a resazurin assay, a common method for measuring metabolic activity of living organisms or indicating liquid pH (Supplementary Figure 4). Results indicate no statistically significant difference between the 0- and 8-hour samples, however, after 12 hours, the color change that we observed on our LADs was correlated with the changes in visible appearance (thickness like yogurt) and odor (sour smell). The colorimetric indications for different incubation times in our experiment are attributed to pH alternations – the pH of the milk shifted from approximately 6 (0, 4, and 8 hours) to approximately 5 (12 hours).

**Figure 5:**
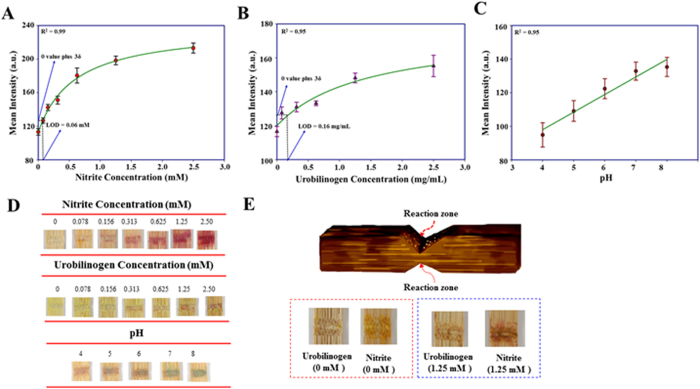
Lignocellulose-based analytical devices for urinalysis. All images of colorimetric results were obtained with a digital camera and analyzed using ImageJ software; these graphs represent the analysis for urinalysis (nitrite, urobilinogen, and pH detection assays). (**A**) The nitrite assay was conducted in human urine, and the establishment of a calibration curve under several concentrations—0, 0.078, 0.156, 0.013, 0.625, 1.25, and 2.5 mM—resulted. (*N* = 6; mean intensity ± S.D. (**B**) The urobilinogen assay was conducted in human urine, and the establishment of a calibration curve under several concentrations—0, 0.078, 0.156, 0.013, 0.625, 1.25, and 2.5 mM—resulted. (*N* = 8; mean intensity ± S.D.). (**C**) The pH detection assay was conducted in human urine, and the establishment of a calibration curve under several concentrations—pH 4, 5, 6, 7, and 8—resulted. (*N* = 4; mean intensity ± S.D.). (**D**) Images display for nitrite, urobilinogen, pH assays. (**E**) Multiple detections of nitrite and urobilinogen assays were measured from human urine. A nitrite detection reagent and an urobilinogen detection reagent were added to different reaction zones on different sides of LADs. The red box indicates that the measurement was in normal human urine sample; the blue box indicates that the measurement was in human urine spiked with 1.25 mM nitrite and 1.25 mM urobilinogen.

In regards to disease detection, we demonstrate rapid urinalysis results for nitrite, urobilinogen, and pH assays using SMLADs. The presence of urine nitrite ions is a crucial index in the diagnosis of urinary tract infections (UTIs), because there should be no nitrite ions in healthy human urine^10^. We serially diluted our nitrite standard solution (10 mM) to five different concentrations in human urine (Fig. 5A). The LOD of our urine nitrite assay was 0.06 mM. Commercial urine dipsticks indicate a positive result through a specific color change if the concentration of urine nitrite is over 0.01 mM^55^. The levels of urine urobilinogen should be less than 1 mg/dL in healthy human individuals^56^. When a person suffers from hemolytic diseases or liver diseases, the levels of urine urobilinogen will significantly increase. We serially diluted an urobilinogen standard solution (20.5 mg/mL) to five different concentrations in human urine (Fig. 5B). The LOD of our urobilinogen assay was 160 mg/dL. It was not quite compatible with the need of current detection criteria. When urine pH is not in the range of 4.5–8.0, a person may suffer from respiratory, metabolic, or renal disorders^57^. We successfully demonstrated that LADs are effective as a pH sensing tool to indicate variations of urine pH (ranging from pH 4.0 to 8.0), and established a standard curve according to the correlation between signal intensity and urine pH (Fig. 5C).

For the development of more diverse applications of LADs, we unveil the capability of multiple detections in one LAD by building two individual reaction zones on opposing sides of bamboo stick substrates (Fig. 5D). Because xylem vessels extend along the longitudinal direction and do not interconnect with each other, no transverse liquid transportation is possible, and LADs can be used to perform multiple detections without cross-contamination between channels. We demonstrate nitrite and urobilinogen assays in human urine using LADs with multiple reaction zones (Fig. 5D). Both assays could display their original colorimetric signals without interference from different adjacent assays.

### HLADs

**Figure 6:**
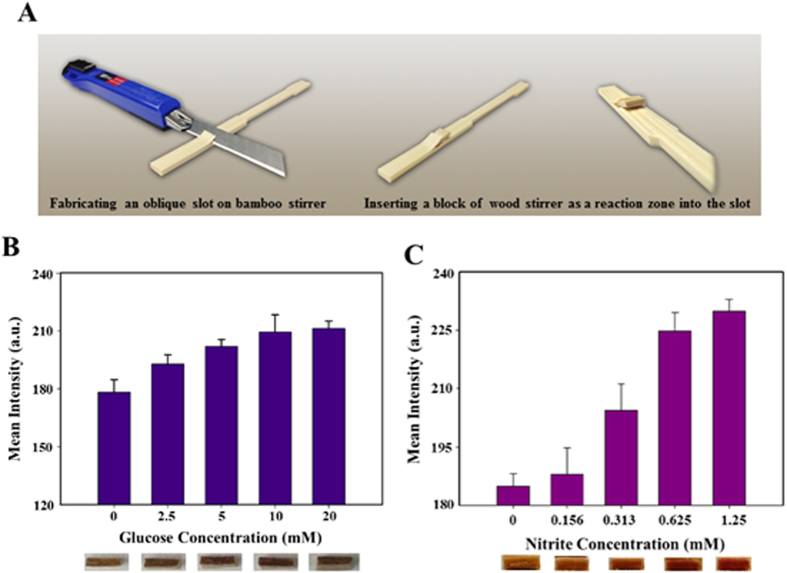
Demonstration of hybrid lignocellulose-based analytical devices. (**A**) Schematic for the fabrication process of HLADs: (I) creating an oblique slot on the bamboo stirrer surface, (II) tailoring wood stirrers into a piece of block whose morphology could be suitably inserted into the oblique slot. Note, the wood block served as a reaction zone for colorimetric assays. (**B**) Glucose assay using HLADs. The standard glucose solution concentrations were prepared in deionized water following the establishment of a histogram under several concentrations—0, 2.5, 5, 10, and 20 mM. (*N* = 6; mean intensity ± S.D.). (**C**) A urine nitrite assay in urine using HLADs. The standard nitrite solution concentrations were prepared in human urine following the establishment of a histogram under several concentrations—0, 0.156, 0.313, 0.625, and 1.25 mM. (*N* = 6; mean intensity ± S.D.) (Photo credits: T.-L. Wang, C.-M. Kuan).

In addition to employing a single material to develop LADs, we explored the development of more complex, multidimensional, and multi-material devices, in which we exploited the separate advantages of bamboo (namely that bamboo wicks liquid at a faster rate than wood) and wood (namely that small pieces of wood provide for more reproducible reaction zones) to make a hybrid device. Figure 6A schematically displays the fabrication process for hybrid LADs, as the combination of wood and bamboos stirrers. We used bamboo stirrers as fluidic channels to transport analytes and a small piece of wood stirrer as a reaction zone. Figures 6B,C display the results of colorimetric glucose and nitrite assays conducted in deionized water and urine, respectively. These images show that the distribution of colorimetric results (purple and deep red color) was relatively uniform on the surface of the wood stirrers (relative to the reaction zone of bamboo).

## Discussion

In this study, we demonstrate the successful development of lignocellulose-based lateral flow platforms for tackling global health issues, specifically food and water safety detections and urinalysis. We used traditional mechanical methods to build our SMLADs and HLADs. The design of LADs does not require additional hydrophobic materials (i.e., wax or polymeric materials) and can resist potential damages from a number of organic solvents. Although a high degree of wicking result irreproducibility appeared when using lignocelluloses, in wood materials in particular, this disadvantage can be significantly improved through appropriate engineering optimization, i.e., optimizing the assay would require an extensive program in engineering development. We performed nitrite assays in deionized water and hot-pot soup as well as a resazurin assay in milk for food safety, a PMS-MTT assay in water for water safety, and nitrite, urobilinogen, and pH assays in human urine for urinalysis using SMLADs. We believe that our device could become a practical platform for reliable and rapid nitrite detection for food safety since i) the detection sensitivity was comparable to current food safety criteria, ii) it performed a stable detection outcome at different days in consideration of the coefficient of variation at different days below 5% (Supplementary Table 1); and, iii) total analysis time was less than 15 minutes. Although the LOD of the bacterial assay is relatively high for the useful detection of bacteria in running water, we provide a method for a low-cost, rapid (less than 1 hour), sufficiently sensitive device that requires no antibodies, enzymes (only a common, visible cell dye), or electricity, and can provide on-site bacterial quantification. Clearly, LADs are comparable with commercial immunochromatographic products (some tests may take roughly several hours) in terms of speed and they provide a comparable LOD (the LODs of commercial immunochromatographic products are in the range of 10^4^–10^7^ cfu/mL without an enrichment step)^58^. Taking food safety analysis a step further, we note that, when determining meat safety, the levels of lactobacillales (lactic acid bacteria) should be less than 10^6^–10^7^ cfu/g or the levels of enterobacteriaceae should be less than 5 × 10^4^–10^6^ cfu/g^59^. These criteria are within the working range of our device (from 1.8 × 10^4^ to 8 × 10^8^), indicating that LADs may be applicable for monitoring meat safety. Although the specific bacterial detection is not currently established, we expect to incorporate an immunochromatographic method or glycan chemistry to develop LAD bacterial selectivity.

For urinalysis, we believe that our device could be an alternative platform for clinical nitrite testing in light of its comparable sensitivity to urine dipstick products. Poor urobilinogen assay sensitivity may be attributable to the following reasons: (i) the original background color from bamboo stirrers abates the LOD; (ii) stirrer-to-stirrer variations expand the deviation between identical measurements; and, (iii) variations in the construction of the reaction zone could increase deviation between measurements. Additionally, the detection of urine pH is one possible extension of LAD applications. Note, a boiling treatment to remove chemical residues on commercial stirrers is enough of a cleaning step for the pre-preparation of LADs used in the above-mentioned assays. As further proof of versatility, we also explore the capability of multiple colorimetric assays using LADs. We believe that this approach could be developed into a practical/commercial device. Not only do we develop SMALDs, but we discuss the design of HLADs that integrate the distinct advantages of bamboo and wood. This platform demonstrates that liquid samples could be rapidly wicked to reaction zones, i.e., pieces of wood, through the vessels of bamboo, and indicates relatively uniform colorimetric results on reaction zones when compared to SMLADs.

More importantly, the lignocellulosic properties of abundant longitudinal microfluidic channels, passive transport with capillary action, and organic solvent resistance support the use of lignocelluloses as prospective engineering materials to advance microfluidic development. We further note that PDMS-based microfluidic systems still must rely on complicated microfluidic channel design and special coating treatments to achieve the above functionalities. Last but not least, we point out that LADs may provide different prospects for low-cost analysis as compared to PADs, as shown in Table 2.

## Methods

### Materials and Chemicals

Stirrers (Supplementary Table 2), deionized water (18 MΩ), polydimethylsiloxane (PDMS) (Sylgard 184, Dow Corning), Whatman qualitative filter paper, No. 1 (GE Healthcare Life Sciences; No. 1001-150), Allura red AC (80%, Sigma Aldrich, St. Louis, MO), Disperse blue 14 (97%, Sigma Aldrich, St. Louis, MO), Fast green (85%, Sigma Aldrich, St. Louis, MO), sodium nitrite (99%, Sigma Aldrich, St. Louis, MO), sulfanilamide (99%, Sigma Aldrich, St. Louis, MO), citric acid (99%, Sigma Aldrich, St. Louis, MO), N-(1-Naphthyl)ethylenediamine dihydrochloride (98%, Sigma Aldrich, St. Louis, MO), bovine serum albumin (BSA) (98%, Sigma Aldrich, St. Louis, MO), tetrabromophenol blue (TBPB) (85%, Sigma Aldrich, St. Louis, MO), 4-Aminoantipyrine (99%, Sigma Aldrich, St. Louis, MO), 4-(Dimethylamino)benzoic acid (98%, Sigma Aldrich, St. Louis, MO), PEG (MW = 35,000 g/mol, Sigma Aldrich, St. Louis, MO), dextrose (Anhydrous, Sigma Aldrich, St. Louis, MO), glucose oxidase (Type X-S, Sigma Aldrich, St. Louis, MO), horseradish peroxidase (HRP) (Type VI-A, Sigma Aldrich, St. Louis, MO), Resazurin sodium salt (80%, Sigma Aldrich, St. Louis, MO), sodium hydroxide (Sigma Aldrich, St. Louis, MO), phenazine methosulfate (PMS) (Sigma Aldrich, St. Louis, MO), 3-[4,5-dimethylthiazol-2-yl]-2,5-diphenyltetrasodium bromide (MTT) (Invitrogen Life Sciences, Carlsbad, CA.), bromothymol blue (95%, Sigma Aldrich, St. Louis, MO), 4-(Dimethylamino)benzaldehyde (99%, Sigma Aldrich, St. Louis, MO), urobilinogen (Santa Cruz Biotechnology, Inc.)

### The Pre-preparation of Lignocellulose-based Analytic Devices

Before the fabrication of LADs, the bamboo and wood stirrers were immersed in 100 °C deionized water for 5 hours. This treatment was carried out to remove unknown chemicals and exclude starch granules from purchased bamboo and wood stirrers. Following this, we placed these stirrers in a 50 °C oven for an 8-hour drying period.

### Verifying Solution Wicking Feasibility for Internal Channels of Lignocelluloses

We coated polydimethylsiloxane (PDMS) [base: curing reagent = 10: 1] around the outside part of LADs with a watercolor paintbrush to decrease the possibility of solutions wicking to the outside part of the device. We then removed gas from the PDMS using a degassing chamber, and placed the PDMS in a 65 °C oven for 1 hour of curing.

### Measuring Color Intensity with ImageJ Software

Images of the reaction zone were captured with a digital camera (EOS 5D Mark III, Canon, Japan), and the obtained images were imported into ImageJ (public software from NIH), which is a suitable image analytical software for analyzing red, green, and blue colors separately. The intensity of each color was measured by selecting the reaction zone and recording the mean intensity of the green channel for our nitrite, PMS-MTT, and urobilinogen assays well as the red channel for our pH detection and glucose assays. This approach has the advantage of reducing the influence of background interference (original lignocelluloses are brown or yellow in color) and increasing result signals between control and experimental values. To analyze the results of our resazurin assay, we individually recorded the values of red, green, and blue colors and relied on Delta RGB calculations as an aide to amplify the value differences between original milk and spoiled milk.

### Analyzing the Results of Each Assay

The values of nitrite, protein, PMS-MTT, urobilinogen, pH, and glucose assays after image processing were normalized by subtracting from 256. We analyzed the results of resazurin assay via Delta RGB calculation (Supplementary Figure 5)^60^.

### Nitrite Assay

The detection principle for nitrite analysis was based on the Griess reaction, which is a universal method for nitrite detection^12^. We immobilized our reagent solution (3 μL of 50 mM sulfanilamide, 330 mM citric acid, and 10 mM N-(1-naphthyl) ethylenediamine dihydrochloride) onto the reaction zone of our LADs with a micropipette; the device was then air-dried for 15 minutes at 25 °C. We immersed the device to a target sample for 7 minutes, and then put the device in an ambient environment for 20 minutes to allow for the chemical reaction process between targeted samples and specific reagents. Afterwards, colorimetric results were recorded using a camera and analyzed with ImageJ software.

### PMS-MTT Assay

The detection principle for our bacterial detection assay was based on the conversion of the oxidation-reduction indicator (MTT) as a means of evaluating bacterial viability. PMS is an intermediate electron carrier that can potentiate tetrazolium (i.e., MTT) reduction^61^. We first immobilized the priming solution (4 μL of 15.63 mM HCl) onto α-cellulose (the reaction zone of SMLADs was filled with 0.023–0.026 g α-cellulose mixture prepared in deionized water) with a micropipette. Then, we immobilized the reagent solution (4 μL of 3.26 mM PMS, 6.03 mM MTT) on top of the priming solution. The device was then air-dried for 2 minutes at 25 °C. We immersed the device to a target sample for 5 minutes, and then put the device in an ambient environment for 35 minutes to allow for the chemical reaction process between targeted samples and specific reagents. At the end of the reaction, we removed the α-cellulose, and added the enhancer (4 μL of 31.25 mM NaOH) to the reaction zone of LADs to amplify the resulting signal of the PMS-MTT assay. Afterwards, colorimetric results were recorded via a camera and analyzed with ImageJ software.

### Resazurin Assay

We first immobilized 4 μL of priming solution (62.5 mM sodium hydroxide solution) onto the reaction zone of our LADs with a micropipette followed by 10 minutes of drying at 25 °C, and then immobilized 2 μL 0.4% w/v resazurin solution on the top of the priming solution. The device was then air-dried for 15 minutes at 25 °C. We immersed the device in a target sample for 7 minutes, and then put the device in an ambient environment for 20 minutes to allow for the chemical reaction process between targeted samples and specific reagents. Afterwards, colorimetric results were recorded via a camera and analyzed with ImageJ software.

### Urobilinogen Assay

The detection principle of urobilinogen was based on the Ehrlich reaction using 4-dimethylaminobenzaldehyde. We first immobilized the reagent solution (3 μL of 0.14 M 4-dimethylaminobenzaldehyde, 2.85 M HCl) onto the reaction zone of our LADs with a micropipette followed by 5 minutes of drying at 25 °C. We immersed the device to a target sample for 20 minutes, and then put the device in an ambient environment for 20 minutes to allow for the chemical reaction process between targeted samples and specific reagents. Afterwards, colorimetric results were recorded via a camera and analyzed with ImageJ software.

### pH Assay

We first immobilized the reagent solution (3 μL of 64.06 mM bromothymol blue, 1.99 mM resazurin sodium salt, 6.4 mM NaOH) onto the reaction zone of our LADs with a micropipette followed by 5 minutes of drying at 25 °C. We immersed the device in a target sample for 7 minutes, and then put the device in an ambient environment for 20 minutes to allow for the chemical reaction process between targeted samples and specific reagents. Afterwards, colorimetric results were recorded via a camera and analyzed with ImageJ software.

### Glucose Assay

The detection principle for glucose assay was based on glucose oxidase- and hydrogen peroxidase (HRP)-mediated couple reaction. Glucose oxidase catalyzed glucose into gluconic acid and hydrogen peroxide; in the presence of HRP, the hydrogen peroxide reacted with colorimetric indicators and generated color oxidation product. We first immobilized the reagent solution [2 μL of 75 U/mL glucose oxidase, 15 U/mL HRP, 2 mM 4-aminoantipyrine, 10 mM 4-(Dimethylamino) benzoic acid, 3% PEG] onto the reaction zone of our LADs with a micropipette followed by 15 minutes of drying at 25 °C. We immersed the device in a target sample for 7 minutes, and then put the device in an ambient environment for 20 minutes to allow for the chemical reaction process between targeted samples and specific reagents. Afterwards, colorimetric results were recorded via a camera and analyzed with ImageJ software.

## Additional Information

**How to cite this article**: Kuan, C.-M. *et al.* Lignocellulose-based analytical devices: bamboo as an analytical platform for chemical detection. *Sci. Rep.* **5**, 18570; doi: 10.1038/srep18570 (2015).

## Supplementary Material

Supplementary Information

Supplementary Video 1

Supplementary Video 2

## Figures and Tables

**Table 1:** Wicking rate for various stirrer types (250 mm^3^).

Stirrer	High (cm)	Time (min) (mean ± S.D.)	Rw (cm/min) (mean ± S.D. (%CV))
WA	2.24	7.22 ± 6.69	0.82 ± 1.00 (121.95)
WB	2.32	12.55 ± 12.75	0.72 ± 0.90 (125.00)
WC	1.4	1.59 ± 2.23	1.97 ± 1.36 (69.03)
WD	1.32	4.17 ± 7.43	1.05 ± 0.85 (80.95)
WE	1.25	2.63 ± 3.07	1.42 ± 1.75 (123.24)
WF	1.96	2.06 ± 2.20	1.69 ± 1.16 (68.64)
BA	1.73	0.30 ± 0.20	7.32 ± 2.93 (40.03)
BB	1.64	0.22 ± 0.09	9.50 ± 6.41 (67.47)
BC	1.64	0.77 ± 1.75	6.90 ± 4.49 (65.07)
BD	2.1	0.25 ± 0.16	11.83 ± 8.28 (69.99)

Wicking rate (Rw) was achieved by recording the amount of time an aqueous dye solution (allura red AC) took to travel the distance—starting from the end of the device that is dipped into solution and continuing away toward the opposite end—such that the overall volume of material that wicks solution was 250 mm^3^. WA-WF represent different types of wood stirrer, BA-BD represent different types of bamboo. (*N* = 18)

**Table 2:** Comparison between LADs and PADs.

	LADs	PADs
(1) Raw material cost	Low	Low
(2) Potential application	Food safety tests, POC diagnostics, bacterial detections (as shown in this research)	Food safety tests, POC diagnostics, heavy metal detections, bacterial detecions^4^,^58^,^62^
(3) Fabrication manner	Mechanical machining (i.e., drilling)	photolithography, CO_2_ laser cutting, printing with polymeric materials or waxes, wax dipping, inkjet etching, plasma treatment, drawing by hand with reagent pens or pencils, melt-and-mold fabrication, and iCVD^4^,^18^,^19^,^20^,^21^,^22^,^23^,^24^,^25^,^26^,^27^,^28^,^29^
(4) The severity of non-uniform colorimetric distribution	Slight	Severe
(5) Long-term monitoring	Possible	Non-uniform colorimetric distribution
(6) Compatibility for organic solvents	Still remaining normal structures	Hydrophobic patterns (polymer material or wax) would be damaged
(7) Device stiffness after immersion	The same	Lower
(8) Biodegradability	Yes	Yes
