# Supplementary material for: Lignocellulose-based analytical devices: bamboo as an analytical platform for chemical detection
Source: Sci Rep. 2015 Dec 21;5:18570. doi: 10.1038/srep18570 (PMC4685277; doi:10.1038/srep18570)
Supplement: Supplementary Information [file srep18570-s1.pdf]

Supplementary Materials

Lignocellulose-based analytical devices: bamboo as an analytical platform for chemical detection

Chen-Meng Kuan[1], Roger L. York[2,3], and Chao-Min Cheng[4,*]

[1]Institute of Nanoengineering and Microsystems, National Tsing Hua University, Hsinchu 30013, Taiwan.

[2]David H. Koch Institute for Integrative Cancer Research, Massachusetts Institute of Technology, 77 Massachusetts Avenue, Cambridge, MA 02139, USA.

[3]Department of Anesethesiology, Boston Children's Hospital, 300 Longwood Avenue, Boston, MA 02115, USA.

[4] Institute of Biomedical Engineering, National Tsing Hua University, Hsinchu 30013, Taiwan.

[*]To whom correspondence should be addressed. Email: chaomin@mx.nthu.edu.tw

Phone number: +886-(3)-516-2402

Fax number: +886-(3)-574-5454

No. 101, Section 2, Kuang-Fu Road, Hsinchu 30013, Taiwan

Keywords: lignocellulose, analytical device, wood, bamboo, bacterial detection

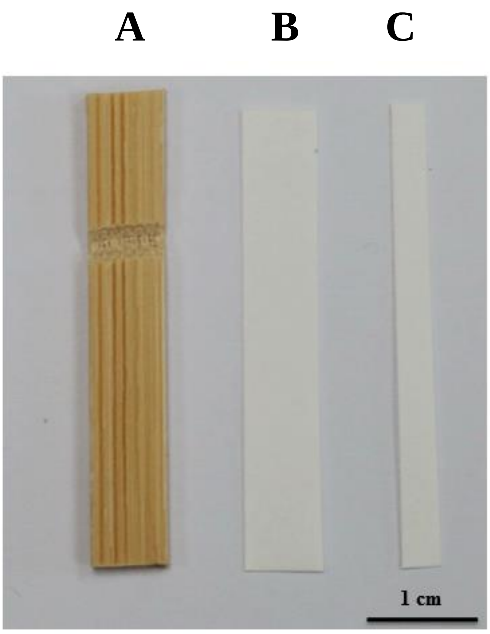

Supplementary Figure 1. Wicking rate comparison between lignocellulose-based analytical devices and paper test strips. (A) LAD: width 0.7 cm; the distance from absorption end to the reaction zone was 3 cm. (B), (C) Paper test strips: width 0.7 cm and 0.3 cm, respectively.

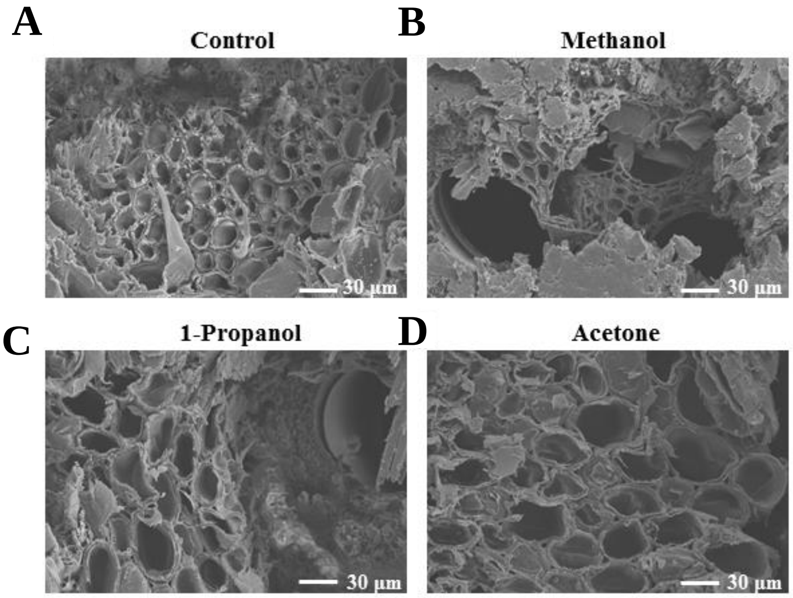

Supplementary Figure 2. The organic resistance test for bamboo stirrers. (A) The SEM image for original purchased bamboo stirrer. The SEM images for bamboo stirrers while undergoing treatment with three different organic solvents: (B) methanol, (C) 1-propanol, and (D) acetone.

A

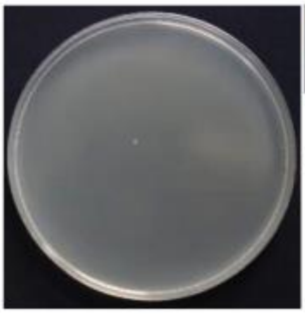
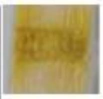

0
cfu/mL

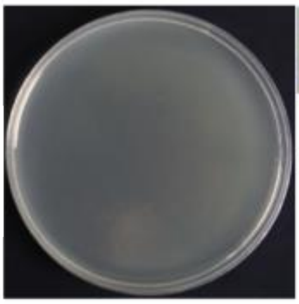
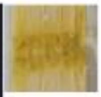

4×10^{3}
cfu/mL

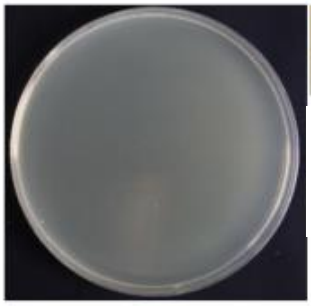
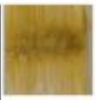

4×10^{4}
cfu/mL

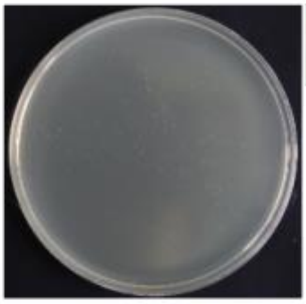
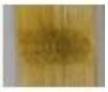

4×10^{5}
cfu/mL

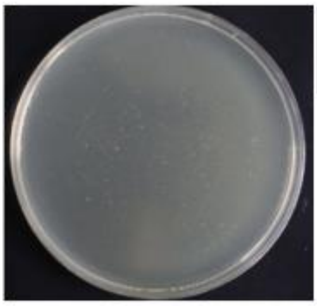
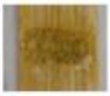

4×10^{6}
cfu/mL

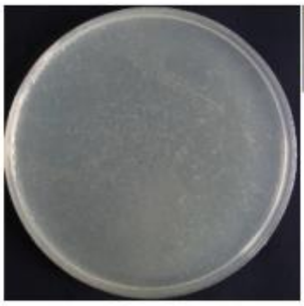
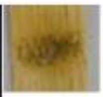

4×10^{7}
cfu/mL

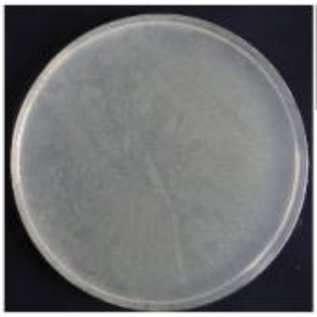
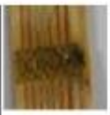

4×10^{8}
cfu/mL

B

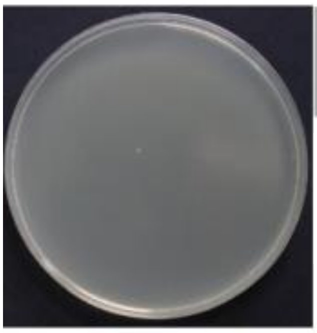
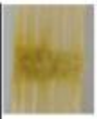

0
cfu/mL

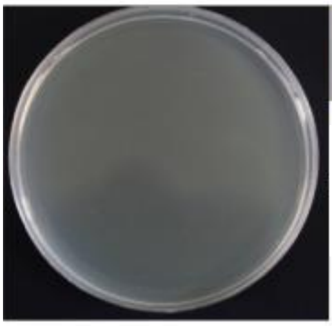
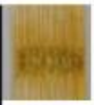

7×10^{3}
cfu/mL

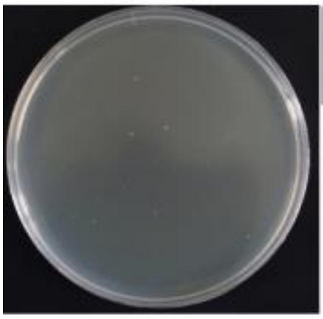
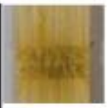

7×10^{4}
cfu/mL

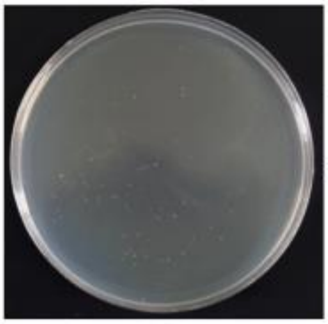
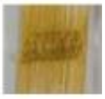

7×10^5 cfu/mL

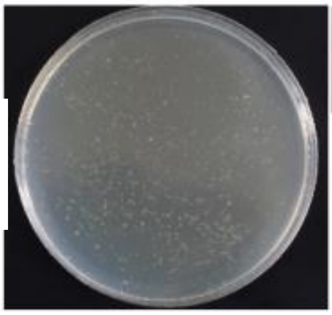
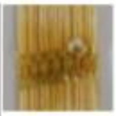

7×10^6 cfu/mL

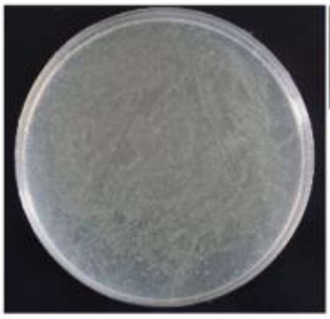
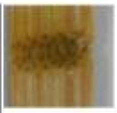

7×10^7 cfu/mL

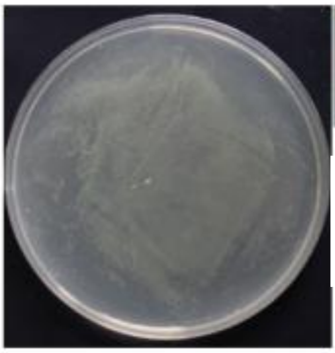
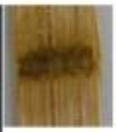

7×10^8 cfu/mL

C

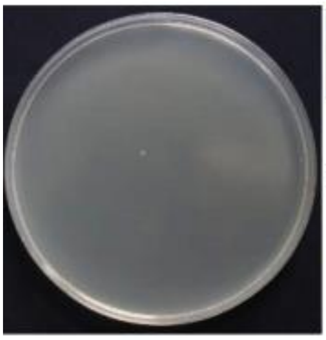
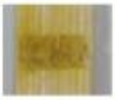

0 cfu/mL

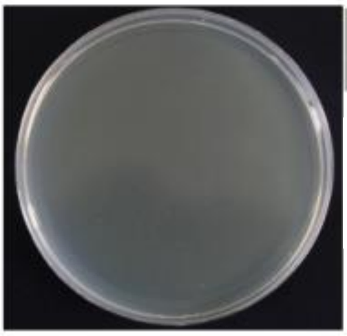
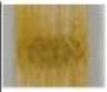

8×10^3 cfu/mL

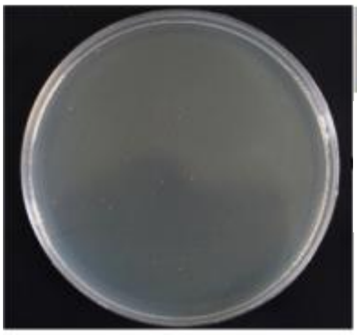
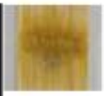

8×10^4 cfu/mL

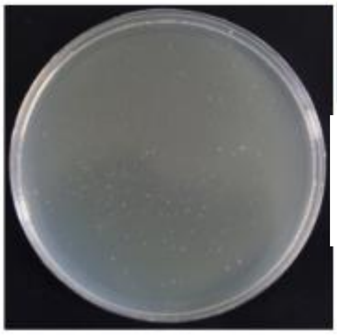
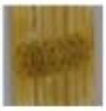

8×10^5 cfu/mL

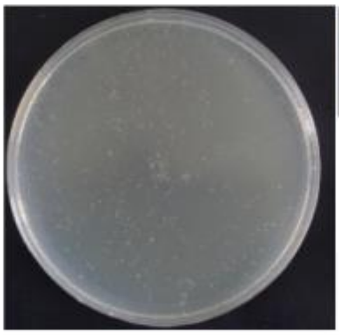
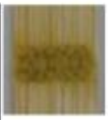

8×10^6 cfu/mL

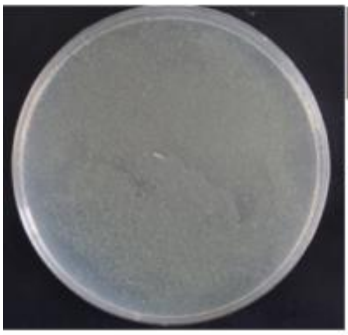
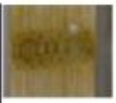

8×10^7 cfu/mL

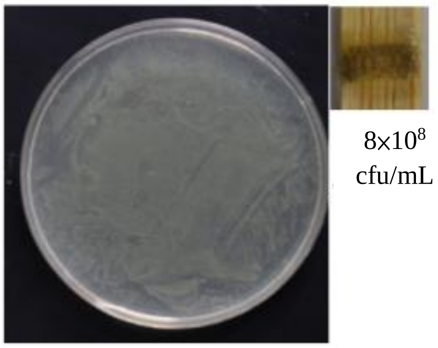

Supplementary Figure 3. The comparison between our bacterial detection method and the agar plate method. (A) The original standard *E. coli* solution sample was 4×10^8, which was serially diluted to the range of 4×10^7-4×10^3 in drinking water. (B) The original standard *E. coli* solution sample was 7×10^8, which was serially diluted to the range of 7×10^7 -7×10^3 in drinking water. (C) The original standard *E. coli* solution sample was 4×10^8, which was serially diluted to the range of 8×10^7 -8×10^3 in drinking water.

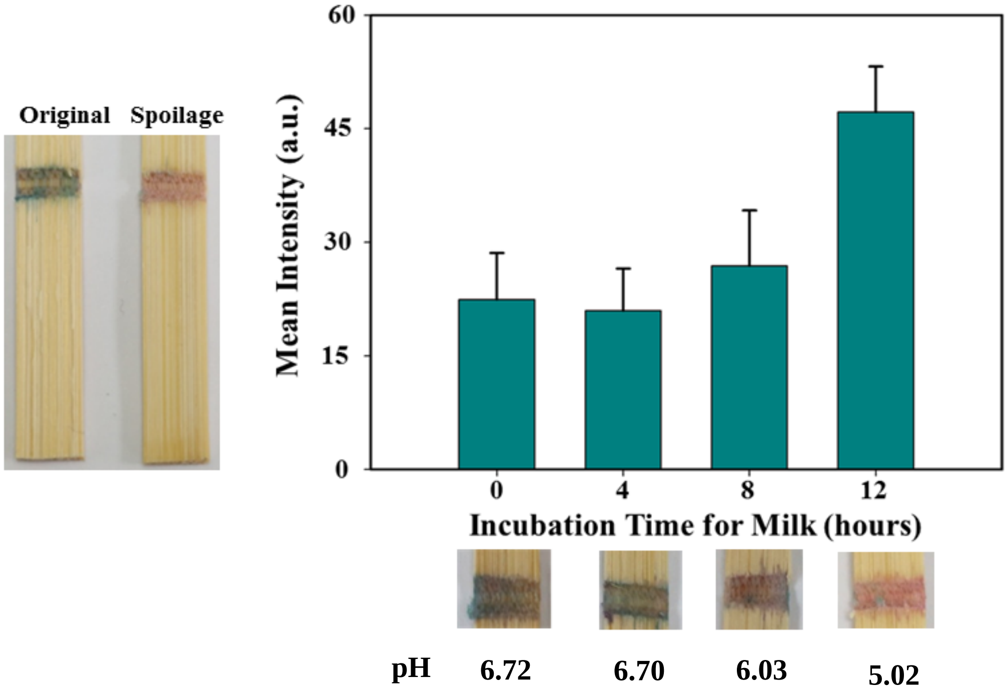

Supplementary Figure 4. Lignocellulose-based analytical devices for the evaluation of milk quality. A resazurin assay for the evaluation of milk quality was conducted at four different incubation times at 37 °C (0, 4, 8, and 12 hours) and the color change noted was from blue (originally purchased fresh milk; 0 hour) to pink (spoiled milk) (N = 8; mean intensity ± S.D.).

$$\Delta RGB = \sqrt{(R_n - R_0)^2 + (G_n - G_0)^2 + (Bn - B_0)}$$

Supplementary Figure 5. Delta RGB calculation. R_0, G_0, and B_0 represent the values of the referenced red, green, and blue colors, respectively – R_0 = 84.60, G_0 = 87.60, and B_0 = 81.39. R_n, G_n, and B_n represent each individual experimental value of red, green, and blue colors.

Supplementary Table 1 Analysis of coefficient of variation for a nitrite assay.

	Concentration (mM)	Intensity (a.u.)	S.D.	%CV
**Within-day variation (day1)**	0.078	83.66	3.41	4.06
	0.156	83.83	2.26	2.7
	1.25	146.72	4.94	3.37
**Within-day variation (day2)**	0.078	80.28	1.22	1.52
	0.156	82.75	2.68	3.24
	1.25	148.06	4.95	3.34
**Within-day variation (day3)**	0.078	84.43	3.14	3.72
	0.156	82.03	2.82	3.43
	1.25	143.08	5.5	3.85
**Between-day variation**	0.078	82.79	3.3	3.99
	0.156	82.87	2.7	3.26
	1.25	145.95	5.55	3.8

Supplementary Table 1 The manufacturing companies of stirrers.

Stirrer	Manufacturing company
WA	Mudanjiang Wanhone Import & Export Co., Ltd.
WB	Starbucks Coffee Company
WC	Li Wei Co., Ltd.
WD	Chian Hung Co., Ltd.
WE	Love Mild Co., Ltd.
WF	Yu Yue Culture Co., Ltd.
BA	Green Bamboo Co., Ltd.
BB	Charng-Chuen Bamboo-Woven Mat Company
BC	Jia Feng Co., Ltd.
BD	Yi Ju Co., Ltd.
